# Superpixel-Oriented Label Distribution Learning for Skin Lesion Segmentation

**DOI:** 10.3390/diagnostics12040938

**Published:** 2022-04-09

**Authors:** Qiaoer Zhou, Tingting He, Yuanwen Zou

**Affiliations:** College of Biomedical Engineering, Sichuan University, Chengdu 610065, China; zhou_qiaoer@outlook.com (Q.Z.); 2019223010062@stu.scu.edu.cn (T.H.)

**Keywords:** label distribution learning, superpixel, skin cancer, segmentation, soft labels

## Abstract

Lesion segmentation is a critical task in skin cancer analysis and detection. When developing deep learning-based segmentation methods, we need a large number of human-annotated labels to serve as ground truth for model-supervised learning. Due to the complexity of dermatological images and the subjective differences of different dermatologists in decision-making, the labels in the segmentation target boundary region are prone to produce uncertain labels or error labels. These labels may lead to unsatisfactory performance of dermoscopy segmentation. In addition, the model trained by the errored one-hot label may be overconfident, which can lead to arbitrary prediction and model overfitting. In this paper, a superpixel-oriented label distribution learning method is proposed. The superpixels formed by the simple linear iterative cluster (SLIC) algorithm combine one-hot labels constraint and define a distance function to convert it into a soft probability distribution. Referring to the model structure of knowledge distillation, after Superpixel-oriented label distribution learning, we get soft labels with structural prior information. Then the soft labels are transferred as new knowledge to the lesion segmentation network for training. Ours method on ISIC 2018 datasets achieves an Dice coefficient reaching 84%, sensitivity 79.6%, precision 80.4%, improved by 19.3%, 8.6% and 2.5% respectively in comparison with the results of U-Net. We also evaluate our method on the tasks of skin lesion segmentation via several general neural network architectures. The experiments show that ours method improves the performance of network image segmentation and can be easily integrated into most existing deep learning architectures.

## 1. Introduction

**Figure 1:**
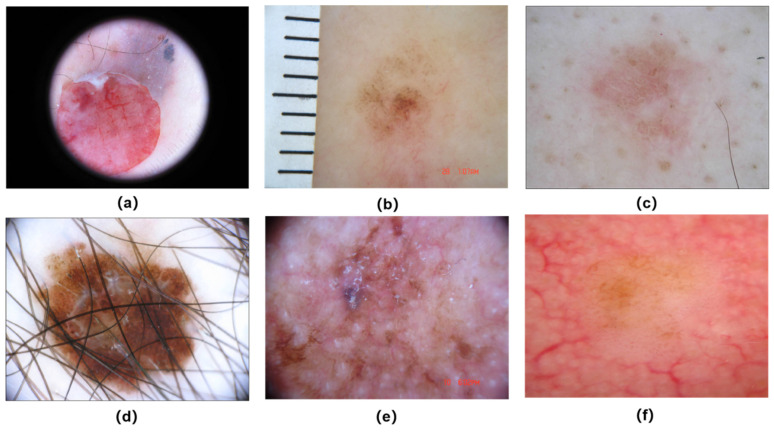
The challenges of automatic skin lesion segmentation from dermoscopy images: (**a**) black frame; (**b**) mark artefact; (**c**) low contrast; (**d**) hair artefact; (**e**) bubbles; (**f**) blood vessels.

Skin cancer is generally caused by the abnormal growth of skin cells, leading to irreversible DNA damage and multiple mutations [1]. The rapid proliferation of abnormal cells may lead to the formation of malignant tumors. There are three main types of skin cancer: basal cell carcinoma (BCC), squamous cell carcinoma (SCC), and melanoma [2]. According to the American Cancer Society’s Cancer Statistics Center, there are about 115,320 new cases and 11,540 deaths in 2021 [3]. The incidence of skin melanoma continues to increase. melanoma is highest degree of malignancy and mortality [4].Early diagnosis and timely treatment are the most effective options for melanoma [5]. Survival varies depending on the diagnosis and cancer margin status is an important indicator of diagnosis. Medical researchers have summed up a variety of clinical diagnosis methods of melanoma based on the color, shape, texture, and visual features of pigmented networks and streaks in the skin lesion area under dermoscopy [6]. For example, ABCD Rule [7], Pattern Analysis [8], Meng’s method [9], and seven-point feature method [10]. With the rapid development of computer-aided diagnosis (CAD) and deep learning technology, there have been various deep learning frameworks to detect and segment skin lesions and achieved good results [11]. Nevertheless, skin lesions segmentation remains a very challenging task because the presence of undesirable artifacts such as (a) black frame; (b) mark artefact; and inherent cutaneous artifacts (c) low contrast; (d) hair artefact; (e) bubbles; (f) blood vessels, as shown in Figure 1.

In addition, due to the inherent visual complexity and ambiguity arising from different skin states, as well as the differences in experience and subjectivity among doctors, we need to consider the possibility of misclassification of semantic segmentation labels, which means that the neural network should be inherently skeptical of the “right answer” to reduce modeling of extreme cases around wrong answers to some extent [12]. Label smoothing is a common solution, which improves the regularization and robustness of the network [13]. Label smoothing gives the label a certain error tolerance probability, but it essentially adds a noise, which does not truly reflect the distribution of labels.

To solve this problem, we propose a superpixel-oriented label distribution learning strategy. The strategy utilizes the structural prior information of dermoscopy to refine the true ground labels and focuses on the underlying feature extraction through the unsupervised simple linear iterative cluster(SLIC) algorithm [14,15], including color space features, location features, and contrast features. Combined with the distance function, the one-hot encoding of the target boundary is converted into a soft probability distribution. Such a label probability distribution can better represent the situation of a sample than one-hot, and a more reliable and interpretable probability distribution is constructed. The design enables us to fully utilize the prior information of medical image segmentation, obtain more informative soft labels and achieve excellent robustness in the segmentation network. We used soft labels to account for the uncertainty in lesion borders’ delineation. We conducted tests and evaluations on PH2 and ISIC 2018 publicly skin cancer image datasets. Experimental results show that this method can significantly improve the performance of the segmentation model.

## 2. Related Works

### 2.1. Segmentation Methods for Skin Lesions

At present, skin lesions segmentation methods are mainly divided into two categories [16,17]. The first category is traditional machine learning image segmentation methods [18], such as edge-based segmentation methods [19], region-based segmentation methods [20],threshold-based segmentation methods [21,22,23], and cluster-based segmentation methods [24,25].

The second category is a segmentation with deep learning. With the wide application of deep learning technology in the fields of image processing and computer vision, the use of deep learning technology to assist clinical diagnosis and decision-making has become a research focus in the field of medical image analysis. Researchers have made important contributions in proposing various deep learning frameworks to detect and segment skin lesions. Based on the exploited deep architecture, we divide these approaches into three groups: Convolutional Neural Network (CNN), Fully Convolutional Network (FCN), and Recurrent Neural Network (RNN).

Convolutional Neural Network (CNN) is a multi-stage, globally trainable artificial neural network model, which can learn abstract, essential and deep features from original data. P.Sabouri et al. [26] proposed a method for lesion boundary detection in clinical images using a 3-layer convolutional neural network (CNN).

The traditional convolutional neural network based segmentation method has high storage cost, low computational efficiency, and limited the size of perception region. Fully Convolutional Network (FCN) is trained end-to-end and pixels-to-pixels, thus solving the problem of spatial resolutions. A study [27] reported segmentation of skin lesions using Fully Convolutional Network (FCN). Yu et al. [28] proposed a 50-layer fully convolutional residual network for skin lesion segmentation, and further improved the accuracy by integrating contextual multi-scale information. Yuan et al. [29] mapped the entire input image into a high-dimensional space through a 19-layer fully convolutional network and proposed a new loss function based on Jaccard distance. Bi et al. [30] proposed a multi-stage fully convolutional network solving the problem of inaccurate segmentation caused by blurred edges or small texture differences in skin lesions. And a parallel fusion method was introduced, which combines the feature information from each segmentation stage to obtain more accurate localization and more detailed segmentation results of skin lesions. U-Net [31] is a semantic segmentation network based on FCN, which is suitable for medical image segmentation. The difference between U-net and FCN is that the up-sampling stage and the down-sampling stage of U-net use the same number of layers of convolution operations, and the skip connection structure is used to connect the down-sampling layer and the up-sampling layer. Thus the down-sampling layer extracts the obtained features can be directly passed to the up-sampling layer, which makes the pixel localization of the U-net network more accurate and the segmentation accuracy higher. SegNet [32] is one of the most popular FCNs, which obtained by modifying VGG-16Net. Kadry et al. [33] employed the VGG-SegNet for segmentation of skin lesions.

Recurrent Neural Networks (RNN) were developed for discrete sequence analysis. RNNs are used to maintain local and global context dependencies. Long Short-Term Memory (LSTM) to learn the spatial dependencies between adjacent pixels. M Attia et al. [34] proposed a deep neural network damage segmentation method using convolutional and recurrent neural networks. Vesal et al. [35] proposed a two-stage segmentation method. The first stage adopted fast RCNN, and the second stage adopted improved U-net and depth extreme value method for segmentation respectively.

In this paper, we demonstrate the improved performance of superpixel-oriented label learning for skin lesion segmentation on U-Net [31] and its derivative network.

### 2.2. Label Smoothing and Knowledge Distillation

Soft labels contain more information than hard labels, leading to better generalization, faster learning, and mitigation of network overconfidence [13,36]. Knowledge distillation [37] and label smoothing [13,38] are two classic ways to obtain soft labels.

Label smoothing is a modification of the loss function that has been shown to be a very effective method for training deep learning networks. Knowledge distillation is a training form using both real labels and soft labels output by the teacher network, which can make up for the lack of supervision signals in classification problems. Knowledge distillation counts the effective information of the data set through the teacher network, retains the correlation information between classes, and removes some invalid redundant information.

Shen et al. [39] proposed that if knowledge distillation does not consider the use of the original data set label items, it is essentially the same as label smoothing. The only difference is that the source of soft supervision information. Knowledge distillation uses a teacher model with fixed weight parameters, which is a dynamic way of monitoring information acquisition. Label smoothing uses artificial smoothing rules.

### 2.3. Label Distribution Learning

Label Distribution Learning(LDL) was proposed by Gengetal in 2010 [40]. Its task is to make the model learn the label distribution of a sample, that is, a probability distribution that builds the mapping relationship from instances to labels. Label distribution learning is used to solve the label ambiguity problem. It uses the feature space of the sample set to construct a label distribution to describe instances [41].

In the existing LDL algorithms, we classify the algorithm construction strategy by label correlation. Geng [42] proposed the BFGS-LLD algorithm, using the maximum entropy model and Kullback-Leibler(KL) divergence. But this algorithm did not consider the relevance of labels. Geng and Hou [43] proposed LDL-SVR, which learns the logit transform of label descriptiveness through SVR. Zheng et al. [44] proposed the LDL-SCL algorithm to explore the correlation between local instances using K-means. Zhou et al. [45] proposed the EDL algorithm, Plutchik’s Emotion Wheel to capture the correlation between emotion labels. Ren et al. [46] proposed LDL-LCLR to exploit global and local label correlations. As can be seen from related works, algorithms using label correlations significantly improve the model’s predictive ability for label distributions.

This subsection introduces deep learning techniques for skin lesion segmentation, label smoothing and knowledge distillation, and related work on label distribution learning. Label smoothing, knowledge distillation, and label distribution learning are all typical methods for generating soft labels. The difference is that label smoothing and label distribution learning use soft labels instead of one-hot labels, while knowledge distillation is a training form using both real labels and soft labels output by the teacher network for training. In addition, label smoothing is artificially set, knowledge distillation is obtained through network inference, and label distribution learning designs a special dedicated algorithm to directly construct label distribution according to the characteristics of the instance. The rest of the paper is as follows: The proposed method is given in Section 3, then the datasets used for experiments and the parameters of the model are given in Section 4. The experimental results are shown in Section 5. The discussion and future scope are given in Section 6.

## 3. The Proposed Method

**Figure 2:**
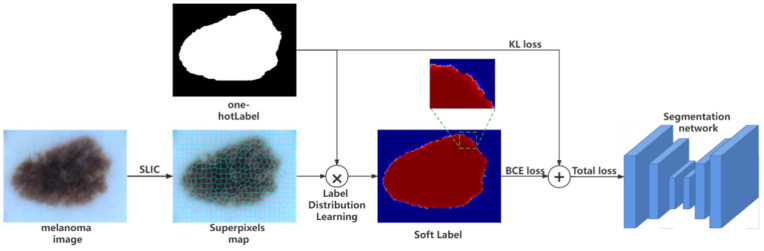
Structure of our proposed method.

An overview of our training pipeline is shown in Figure 2. The model consists of two stages, below we first introduce superpixel-oriented label distribution learning in Section 3.1. We then introduce various deep learning networks trained with collaborative soft and hard labels in Section 3.2.

### 3.1. Superpixel-Oriented Label Distribution Learning Method

Superpixel-oriented label distribution learning enables us to fully exploit prior information for medical image segmentation. Superpixelation was proposed by Xiaofeng Ren in 2003 [47]. Superpixels cluster image pixels based on the local structural features of the image and the spatial relationship between pixels, and generally do not destroy the boundary information of objects in the image. This method aggregates image pixels into a series of adjacent pixel blocks with similar features such as color, brightness, texture, etc., which enhances the edge features between superpixels. We use a simple linear iterative clustering algorithm (SLIC) method [14] to generate these superpixels while obtaining corresponding soft labels according to the mapping relationship between the dermoscopy boundaries (hard labels) and the locations of superpixel blocks. Specifically, to exploit image structure priors and spatial correlations in labels, we first compute superpixels of training images, creating visually meaningful instances. Then, each superpixel is compared with the boundary in the hard label. If it intersects with the boundary, the corresponding label of the superpixel block is reassigned through the distance mapping formula. If it does not intersect with the boundary, the label remains unchanged. Then, the soft and hard labels are used jointly to train the segmentation network. We can also think of this approach as a kind of labeled data augmentation.

**Figure 3:**
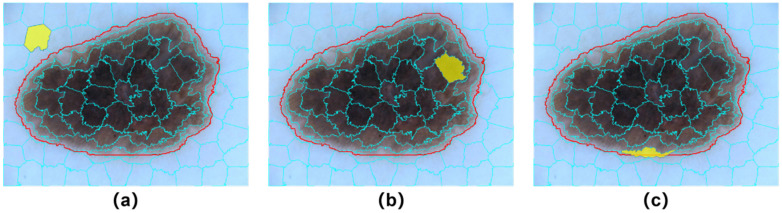
Illustration of relations between the boundary and a superpixel block. (**a**) superpixel is outside of the boundary; (**b**) superpixel is inside of the boundary; (**c**) superpixel intersects with the boundary.

As shown in Figure 3, we treat superpixel intersection regions as uncertain label regions and reconstruct soft labels through distance mapping with ground truth annotations. The yellow area represents the current superpixel area, the red line represents the mask lesion boundary, and the blue line represents the superpixel segmentation line. There are two types of relationships between superpixel regions and boundaries: (a,b) disintersect and (c) intersect, as shown in Figure 3. The distance to the boundary is calculated for each pixel in the superpixel region that intersects the boundary, and then the membership probability [0, 1] of the pixel in the lesion and normal skin regions is generated according to the distance mapping formula.

We define the distance Formulas (1):

(1) $$d\left(x,x_{I}\right)=\min\left(\left|x-x_{I}\right|\right)\;\;for\;all\;\;x_{I}\;\in \;\partial$$

$d\left(x,x_{I}\right)$ aims to find a point $x_{I}$ that belongs to the boundary ∂Ω, and this point $x_{I}$ must be the closest point to *x* among all the points on the boundary, so as to calculate the distance between them.

Formulas (2)–(4) reflect the positional relationship between the point and the contour [48]. We set the value to less than 0.5 when the point is outside the contour. Return a value greater than 0.5 when the point is inside, or 0.5 if the point is on the contour. Therefore, as the distance increases, the pixels inside the boundary are closer to 1 (corresponding to white), and the pixels outside the boundary are closer to 0 (corresponding to black).

(2) $$f\left(x\right)=\frac{1}{2}\;\;if\;\;\;x\in \partial \Omega$$

(3) $$f\left(x\right)=\frac{1}{2}\left(1+\frac{d\left(x,x_{I}\right)}{1-d\left(x,x_{I}\right)}\right)\;\;if\;\;\;x\in \partial \Omega^{+}$$

(4) $$f\left(x\right)=\frac{1}{2}\left(1+\frac{d\left(x,x_{I}\right)}{1+d\left(x,x_{I}\right)}\right)\;\;if\;\;\;x\in \partial \Omega^{-}$$

**Figure 4:**
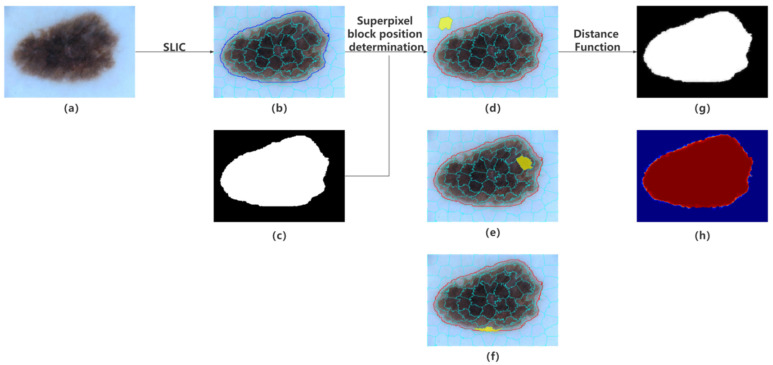
Pipelines for generating soft labels. (**a**) Original image. (**b**) Superpixels map. (**c**) Hard Ground truth. (**d**–**f**) Illustration of relations between the boundary and a superpixel block. (**g**) Soft Ground truth. (**h**) Colormap of soft Ground truth.

Figure 4 shows the pipelines for generating a soft label. Considering the computational cost and soft label quality, we set different total numbers of superpixels to search for optimal parameters.

### 3.2. Deep Learning Networks

The main purpose of the method in this paper is to measure the similarity between the expression of the sample and the label, and convert the data label into a soft probability distribution. The relationship between labels and samples is captured by the superpixel segmentation method, and the generated superpixel blocks are regular in shape and uniform in size, and relatively completely retain the texture feature information in the skin lesion area. This measure of similarity is then added to the one-hot label. Therefore, when the network is combined with soft label training, the information learned is more abundant.

The concept of collaborative label learning originates from knowledge distillation [37]. Inspired by knowledge distillation, we define the Total Loss Formula (5) as the weighted average of the cross-entropy corresponding to soft labels and hard labels.

(5) $$Total\;loss=\alpha L_{CE}+\beta L_{KL}$$

The first term $L_{CE}$ is the error between the model result and the real value, and the second term $L_{KL}$ is the error between the model result and the soft label. In joint training, we search for the network parameters with the best segmentation effect by adjusting the weights of the loss function.

We adopt *KL* divergence to set the soft label loss function Formula (6) and *CE* (cross entropy) to set the hard label loss function Formula (7):

(6) $$L_{KL}(p_{soft}||q)=\frac{1}{N}\sum_{x=1}^{N}p_{soft}\left(x\right)\cdot \left(log\;p_{soft}\left(x\right)-log\;q\left(x\right)\right)$$

(7) $$L_{CE}\left(p_{hard},q\right)=-\frac{1}{N}\sum_{x=1}^{N}p_{hard}\left(x\right)\cdot log\;q\left(x\right)$$

where $p_{soft}$ is the predicted probability of soft label, $p_{hard}$ is the predicted probability of hard label, p(x) is the probability value for the *x*-th pixel, and q(x) is the distribution of the prediction model for the *x*-th pixel.

## 4. Experiments

To validate the generality of our approach, we trained a variety of neural network architectures on PH2 and ISIC 2018 datasets. In particular, we trained three different neural network architectures: U-Net [31], U-Net++ [49], AttU-Net [50]. We observe that choosing the correct superpixel parameters and soft label weights is one of the key parameters determining the segmentation performance. Therefore, we propose 3 steps to select the best parameters and evaluate the skin lesion segmentation performance:

(1) Estimate the best value of N_segment by the u-net network trained on PH2;
(2) The optimal weights for soft and hard labels are estimated by the u-net network trained on the ISIC2018 and PH2 datasets;
(3) The proposed method is evaluated for segmentation performance on the skin lesion segmentation task using general neural network architectures, including U-Net [31], U-Net++ [49], AttU-Net [50], using the best parameters.

### 4.1. Datasets

The ISIC2018 dataset [51] contains 2594 dermatological images raw images with corresponding binary label images, including actinic keratosis and intraepithelial neoplasia, benign keratosis, basal cell carcinoma, squamous Skin diseases such as cell carcinoma, dermatofibroma, melanoma, mole, and vascular lesions. The PH2 dataset [52] has 200 images, of which 160 are moles (atypical and common) and 40 are melanomas.

Ithe superpixel-oriented label distribution learning part, all images are resized to 500 × 375 in order to reduce the computational cost. From the total number of superpixels 500 to 1500, every 500 is an interval. Different quality soft labels are generated through different parameters to form different label sets. Label sets include soft labels and hard labels. In order to reduce the computational cost, we take the superpixel parameters on the ph2 dataset and directly apply them to the isic2018 dataset.

Before training the segmentation network, the dataset were splited into a training set (80%) and a test set (20%), respectively. To increase the number of training sets, we applied random vertical and horizontal flips, random rotations as data augmentation.

### 4.2. Evaluation Indicators

We evaluated the performance of the segmentation by precision, sensitivity, intersection-over-union (IOU), dice coefficient, average surface distance (ASD), and hausdorff distance (HD) as evaluation metrics. The formulas are shown in Equations (8)–(13) below. Our performance metrics rely on calculation of the true negative (TN), false negative (FN), false positive (FP), and true positive (TP) regions of the segmented image which are calculated by comparing regions of the skin lesion which are either correctly or incorrectly classified as being skin lesion or background regions. “↓” means smaller is better and “↑” means bigger is better.

Precision indicates the proportion of the predicted positive samples, defined in Equation (8). Sensitivity, Same as recall, indicates good performance in segmentation which implies all the lesions were segmented successfully, defined in Equation (9).

(8) $$Precision↑=\frac{TN}{TP+FP}$$

(9) $$Sensitivity↑=\frac{TP}{TP+FN}$$

The IOU indicator, also known as the Jaccard index, is one of the most commonly used metrics in semantic segmentation. Iou is the area of overlap between predicted segmentation and label divided by the difference between predicted segmentation and label Joint area, defined in Equation (10).

(10) $$IOU↑=\frac{TP}{TP+FP+FN}$$

The Dice coefficient is defined as the intersection of two times divided by the sum of pixels, also called F1 score, defined in Equation (11).

(11) $$Dice↑=\frac{2*TP}{\left(2*TP+FP+FN\right)}$$

The ASD is the average surface distance of all point sets of the prediction. It is also an evaluation metric in the medical image segmentation competition CHAOS. It is defined in Equation (12).

(12) $$ASD↓=\frac{1}{S\left(A\right)+S\left(B\right)}\left(\sum_{S_{A}\in S\left(A\right)}d(S_{A},S\left(B\right)\right)+\sum_{S_{B}\in S\left(B\right)}d(S_{B},S\left(A\right)))$$

where *S*(*A*) represents the surface voxel in the *A* set *d*(*v*, *S*(*A*)) represents the shortest distance from any voxel to *S*(*A*).

HD (Hausdorff distance) is used for segmentation indicators, mainly to measure the segmentation accuracy of boundaries. In the actual calculation, we do not select the maximum distance, but arrange the distances from large to small, and take the ranking as 5% of the distance. The purpose of this is to exclude the unreasonable distance caused by some outliers and maintain the stability of the overall value. Therefore, it is also called HD95. Dice is more sensitive to the inner filling of the mask, while Hausdorff distance is more sensitive to the segmented boundary. It is defined in Equation (13).

(13) $$d_{H}\left(A,B\right)↓=\max\left\{d_{AB},d_{BA}\right\}=\max\left\{\underset{a\in A}{\max}\;\underset{b\in B}{\min}\;d\left(x,y\right),\underset{b\in B}{\max}\;\underset{a\in A}{\min}\;d\left(x,y\right)\right\}$$

where d(x,y) represents the distance paradigm between point sets *A* and *B*.

### 4.3. Implementation Details

As far as the field of deep learning is concerned, the amount of data in the ISIC 2018 and PH2 datasets used in this paper is very small. Because U-Net [31] has good performance in small sample data sets, We choose U-Net to split the network for experiments. The network training algorithm used in the experiment is an Adam [53] algorithm with a faster convergence speed. training The process takes 20 Epoch training, the Batchsize is set to 40, and the learning rate (Lr) is 0.001. We used an Early Stopping strategy to prevent overfitting (Patience is 50epochs). All experiments are done on a cloud computing platform using 2 NVIDIA TeslaP4024G GPUs and a 14-core CPU configuration. The experiments use python coding and the PyTorch framework [54].

We observe that choosing the correct superpixel parameters and soft label weights is one of the key parameters determining the segmentation performance, and superpixels of different sizes describe different levels of image features. We first establish a baseline for the segmentation network, which is trained without soft labels. The optimal parameters of the superpixel-oriented label learning method are then searched on the PH2 dataset.

**Figure 5:**
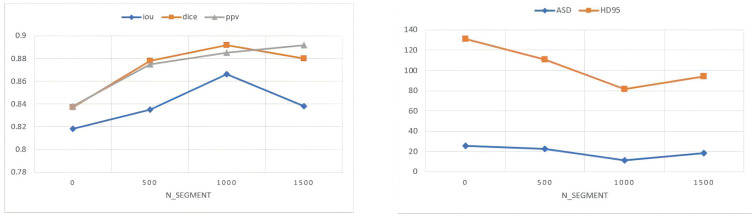
Performances of our method with different values of N_Segment on PH2 dataset. The HD95 and ASD are in mm.

Figure 5 shows the performance impact of our method over different ranges of superpixel numbers. In order to control the variables, we set the value of α, β are both 1. The total number of superpixels(N_SEGMENT) ranges from 0 to 1500, and the sampling interval is 500. The total number of superpixels is negatively correlated with the superpixel block size. If the total number of superpixels is small, information will be lost, and if the superpixel block is too large, it is easy to generate soft labels with incorrect information. If the total number of superpixels is too large, it will not only increase the computational cost but also show a significant downward trend in the segmentation performance after reaching the peak. We can reasonably speculate that as the number of superpixels increases, the size of the superpixel block decreases, and the number of superpixels that interact with the boundary decreases, thus hindering the generation of soft labels. According to the statistics, the size of the superpixel parameters is positively proportional to the time required. The number of superpixels is 500 on average took 8.4 s per image; the number of superpixels is 1000 on average took 15.6 s per image; the number of superpixels is 1500 on average took 24 s per image. It can be seen from the figure that when the total number of superpixels (N_Segment) is 1000, the performance of iou, dice, ASD and HD95 is the best. As the total number of superpixels increases, the performance of PPV (precision) is further improved, but other indicators show a downward trend. Considering the best overall segmentation performance, we set the total number of superpixels to 1000 for experiments on the ISIC 2018 dataset.

**Figure 6:**
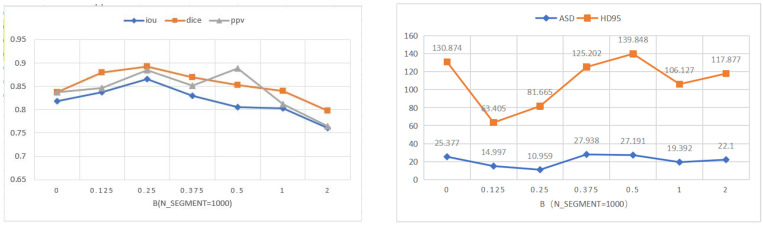
Performances of our method with different values of β on PH2 dataset.Set the horizontal axis to Log Scale.

**Figure 7:**
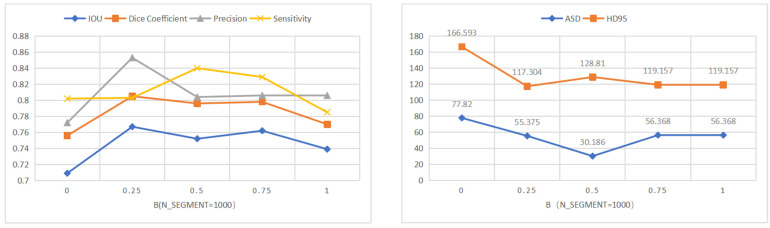
Performances of our method with different values of β on ISIC 2018 dataset.

Then, we will establish a baseline on the PH2 and ISIC 2018 datasets. In order to achieve the best segmentation effect for collaborative label learning, we adjust α, β to control the contribution of hard and soft labels in training. In order to facilitate quantitative analysis, we set a = 1, and b takes the value from 0–2. Figure 6 and Figure 7 show the performance of the indicators IOU, dice, precision, sensitivity, ASD and HD95 under different values of β. When β = 0, it is the baseline of our method. An improvement in segmentation performance can be observed when β is increased. However, when β continues to increase, the overall segmentation performance starts to degrade. When β = 0.5, the overall performance of precision is the best on the PH2 dataset. When β = 0.25, the overall performance of precision is the best on the ISIC 2018 dataset. In the following comparison with other segmentation networks on the ISIC 2018 dataset, we will take the value of β as 0.25.

## 5. Results

**Figure 8:**
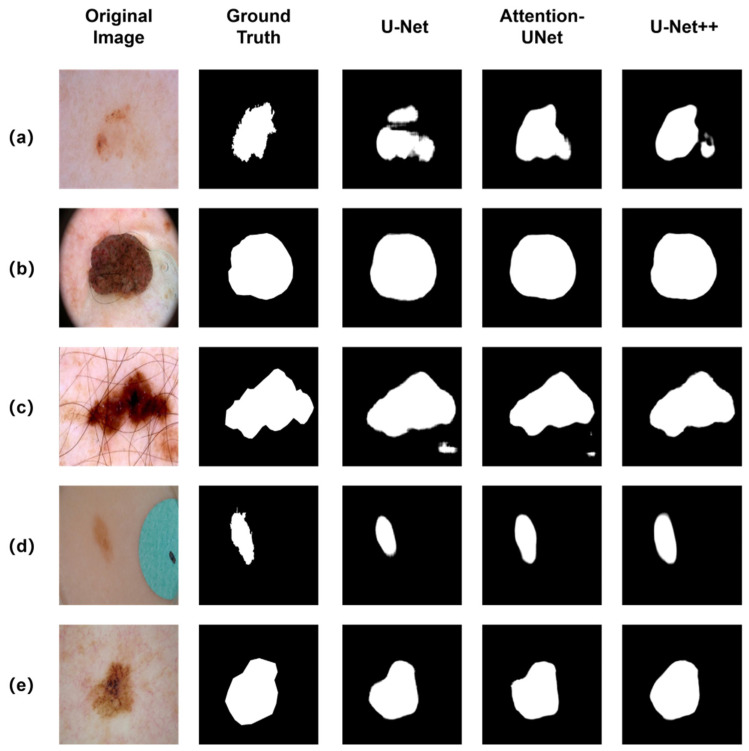
Visual comparison of lesion segmentation results produced by different segmentation network architecture with superpixel-oriented label distribution method. (**a**) low contrast; (**b**) black frame and bubbles; (**c**) hair artefact; (**d**) color illumination; (**e**) blood vessels.

Table 1 summarizes the segmentation performance metrics for three segmentation networks on the ISIC 2018 dataset. We evaluate segmentation performance along with four key metrics: the Dice coefficient, the intersection-over-union (IOU) score, precision, and Sensitivity. In all cases, training model with superpixel-oriented soft labels improves the segmentation in most metrics when compared to training the same model as baseline. As is to be expected, the improvement of segmentation performance with superpixel-oriented Label distribution learning is evident and clearly indicated. The Visual comparison of lesion segmentation results produced by different segmentation network architecture with superpixel-oriented label distribution method are shown in Figure 8.

We also compare our methods to other state-of-the-art methods that use the same datasets, shown in Table 2. Our approach can be used as a data augmentation method of label for existing and future segmentation methods that use neural networks to provide additional segmentation improvement. The ISIC 2018 dataset has 2594 dermatological images, and the network has enough data to learn the features of dermatological images. Therefore, the improvements gained from our method is also not as large as the small dataset (PH2).

## 6. Discussion

In this work, we propose a superpixel-oriented label distribution learning method to generate probability distribution labels. Using the framework of knowledge distillation, combined with soft label and hard label training mode. Our method applies the SLIC algorithm to capture the relationship between instances and labels and generate soft labels with structural priors. The soft label can be regarded as a regularization term during training to constrain the distribution of parameters in the network. The original label is a discrete hard labels. The soft labels not only give the correlation between pixels, but also can be regarded as a data augmentation in the label space. Experiments on PH2 and ISIC 2018 datasets proved the increased segmentation performance of the superpixel-oriented distribution learning method on several popular deep learning models such as U-Net [31], AttU-Net [50] and U-Net++ [49]. The segmentation performance improvement of the proposed method depends on the selection of the correct hyper-parameters, which indicates potential limitations to generalize to new data sets and tasks. Future research directions can explore ways to alleviate this dependence. In addition, we can make further improvements in the following areas:

(1) Denoising of skin disease data [23]. When collecting dermoscopic images, differences in skin texture, the appearance of symptoms, collection operation procedures, and collection environments of different patients will result in the uneven quality of dermoscopic images. removing the black frame and hair noise, more accurate lesion information can be obtained.
(2) K-fold cross-validation [61]. Cross-validation is a method for model selection by estimating the generalization error of the model. It has universal application and is easy to operate.
(3) Loss function. A suitable loss function in image segmentation can help the model to converge faster and better. We can try several common loss functions, such as Dice Loss [62], Tversky loss [63], and Focal Loss [64], etc.
(4) Fully Connected CRFs [65]. Fully connected CRFs is an image post-processing method commonly used in deep learning image segmentation applications. It is an improved mode of CRFs, which can combine the relationship between all pixels in the original image to process the classification results obtained by deep learning, optimize Classify rough and indeterminate markers in images, correct finely misclassified regions, and obtain finer segmentation boundaries.

## Figures and Tables

**Table 1:** Performance analysis and comparison of Our method with the baseline on ISIC 2018 dataset.

Segm. net.	Parameters	Sensitivity	Precision	Dice Coefficient	IOU
U-Net [31]	α=1 β = 0	0.708	0.779	0.647	**-**
	α = 1 β = 0.25	**0.796**	**0.804**	**0.840**	0.752
AttU-Net [50]	α =1 β = 0	0.717	0.787	0.665	**-**
	α =1 β = 0.25	**0.766**	**0.91**	**0.828**	0.761
U-Net++ [49]	α =1 β = 0	0.786	**0.908**	0.809	0.728
	α =1 β = 0.25	**0.840**	0.903	**0.868**	**0.825**

**Table 2:** A comparison between our method (approach with best results) and the state of the art on the same datasets.

Model	ISIC 2018				Model	PH2			
	Dice	IOU	Precision	Sensitivity		Dice	IOU	Precision	Sensitivity
AttR2U-net [55]	0.734	0.581	0.822	0.726	FCN [56]	0.890	0.802	0.877	0.903
ResUnet++ [57]	0.855	0.813	0.867	0.880	SegNet [34]	0.893	0.807	0.923	0.865
DeepLab V3+ [58]	0.871	0.798	0.880	**0.906**	U-Net [31]	0.876	0.779	**0.945**	0.816
DoubleU-Net [59]	**0.896**	0.821	**0.945**	0.878	FrCN [60]	**0.917**	0.847	0.899	0.937
Our method	0.868	**0.825**	0.903	0.840	Our method	0.892	**0.866**	0.885	**0.976**

## Data Availability

No new dataset was generated from this study. We utilized the following two public datasets in this study: https://www.fc.up.pt/addi/ph2%20database.html (accessed on 6 March 2022) and https://challenge2018.isic-archive.com/task1/ (accessed on 6 March 2022).
